# Comparison of the incidence of extended-spectrum βlactamase-producing *Enterobacterales* (ESBL-PE) before, during, and after the COVID-19 pandemic in a tertiary hospital in indonesia

**DOI:** 10.1017/ash.2025.109

**Published:** 2025-09-03

**Authors:** Fadrian Fadrian, Linosefa Linosefa, Armen Ahmad, Rohayat Bilmahdi Simanjuntak, Vidola Yasena Putri

**Affiliations:** 1 Department of Internal Medicine, Division of Tropical and Infectious Disease, Faculty of MedicineUniversitas Andalas, Dr. M. Djamil General Hospital, Padang, Indonesia; 2 Department of Clinical Microbiology, Faculty of MedicineUniversitas Andalas, Padang, Indonesia; 3 Indonesian Society of Infection Control (INASIC), West Sumatera Region, Indonesia; 4 Faculty of MedicineUniversitas Andalas, Padang, Indonesia

**Keywords:** COVID-19, ESBL, MDRO

## Abstract

**Objectives:** The COVID-19 pandemic has become a serious threat to global health. Current research shows that COVID-19 causes an increase in the incidence of multidrug-resistant organisms (MDRO) due to excessive use of antibiotics during COVID-19 [1,2]. Extended-spectrum β lactamase-producing *Enterobacterales* (ESBL-PE), especially *Escherichia coli*-producing ESBL (Eco-ESBL) and *Klebsiella pneumoniae*-producing ESBL (Kp-ESBL) are pathogens of current concern due to their potential for rapid spread in communities and healthcare [3]. Based on antibiogram data from Dr. M. Djamil General Hospital Padang in 2022, the incidence of MDRO in the inpatient, outpatient, and intensive care units was mostly caused by Kp-ESBL (12,7%), followed by Eco-ESBL (11,9%) [4]. This study aims to compare the incidence of MDRO caused by Eco-ESBL and Kp-ESBL before, during, and after COVID- 19. **Methods:** This research constitutes a retrospective descriptive study conducted at Dr. M. Djamil General Hospital Padang during three distinct periods: before, during, and after the COVID-19 pandemic. The population of this study was the results of all cultures from all specimen examinations that produced ESBL. Two thousand and seventeen samples were taken from the population that met the inclusion and exclusion criteria using the total sampling technique. **Results: Comparison of the incidence** showed that Eco-ESBL has an increased risk of incidents after the pandemic by 1.41 times compared to before the pandemic, while the risk of incidence during the pandemic does not show a significant relationship (p=0.63, p>0.05). In contrast to Kp-ESBL, there is a decrease in the risk of incidence after the pandemic by 0.62 times compared to before the pandemic (p<0.05), while the risk of incidence during the pandemic also does not show a significant relationship (p=0.63, p>0.05). **Conclusion:** There is a significant risk of incidence of MDRO caused by Eco-ESBL and Kp-ESBL after the pandemic compared to before the pandemic COVID-19.

